# Comparative Effectiveness of Short-Course Versus Standard-Course Empiric Antibiotic Therapy for Acute Bacterial Meningitis: A Systematic Review

**DOI:** 10.7759/cureus.114618

**Published:** 2026-08-16

**Authors:** Amit Agrawal, Dhaval Shukla, Suresh Thanneeru, Pramod K Sharma, Roshan Chanchlani, Amber Kumar, Rajnish Joshi, Jitendra Singh, Charushila Rukadikar

**Affiliations:** 1 Neurosurgery, All India Institute of Medical Sciences, Bhopal, IND; 2 Neurosurgery, National Institute of Mental Health and Neurosciences (NIMHANS), Bangalore, IND; 3 Pediatric Surgery, All India Institute of Medical Sciences, Bhopal, IND; 4 Pediatrics, All India Institute of Medical Sciences, Bhopal, IND; 5 Internal Medicine, All India Institute of Medical Sciences, Bhopal, IND; 6 Translation Medicine, All India Institute of Medical Sciences, Bhopal, IND; 7 Physiology, All India Institute of Medical Sciences, Gorakhpur, IND

**Keywords:** antibiotic treatment, bacterial meningitis, empiric antibiotics, meningitis, short-course antibiotic therapy

## Abstract

Acute bacterial meningitis (ABM) remains a significant cause of mortality and long-term neurodevelopmental morbidity in children. While standard treatment often involves 14-21 days of antibiotic therapy, the optimal duration of empiric treatment remains debated. This systematic review aimed to compare the effectiveness and safety of short-course empiric antibiotic treatment (<10 days) versus standard-course treatment (14-21 days) in reducing mortality and morbidity in patients with suspected ABM.

We followed the Preferred Reporting Items for Systematic Reviews and Meta-Analyses (PRISMA) guidelines. A systematic search was conducted across electronic databases, including EMBASE, MEDLINE, SCOPUS, the Cochrane Library, and clinical trial registries. We included randomized controlled trials (RCTs) and observational studies in which the authors reported and compared antibiotic treatment durations for bacterial meningitis. Data on antibiotics, doses and treatment durations, mortality, neurological sequelae (including hearing loss, developmental delay, and motor deficits), and hospitalization outcomes were extracted and analyzed.

Eight studies, all of which were RCTs, involving 1,562 patients were included. Seven studies included pediatric populations, while one included neonates. Short-course therapy (4-10 days) demonstrated clinical outcomes comparable to those of standard-course therapy (14-21 days), with respect to the primary outcomes. No significant differences were observed in mortality rates, the incidence of hearing loss, or long-term neurological sequelae between the treatment groups. Shorter antibiotic courses were associated with a significantly reduced duration of hospitalization (p < 0.05) and lower rates of nosocomial infections. Older studies (pre-2011) used ceftriaxone monotherapy, whereas more recent studies used combination therapy with ceftriaxone and vancomycin.

Short-course antibiotic therapy is a safe and effective alternative to standard regimens for pediatric and neonatal bacterial meningitis. Shorter treatment durations may reduce healthcare costs, hospital-acquired infections, and cumulative antibiotic exposure. However, large, multicenter RCTs are needed to further validate combination therapy protocols and support the development of standardized, evidence-based guidelines.

## Introduction and background

Acute bacterial meningitis (ABM) can lead to residual neurodevelopmental sequelae in the pediatric population and may be associated with permanent disabilities [1]. Globally, ABM remains a major cause of pediatric mortality, particularly in low- and middle-income regions, where the burden of disease is highest [2]. ABM can result in complications, including neurological damage, hearing loss, and developmental delays. Early diagnosis and appropriate treatment are crucial for improving outcomes and preventing complications. *Streptococcus pneumoniae*, *Neisseria meningitidis*, and *Haemophilus influenzae *are among the most common organisms causing bacterial meningitis [3,4]. Empiric antibiotic therapy is typically initiated based on the suspected causative organisms and local antimicrobial susceptibility patterns or treatment guidelines. Treatment may involve a single antibiotic or a combination of antibiotics. The antibiotic regimen is usually adjusted according to culture results after 48-72 hours. This transition from broad empiric coverage to pathogen-directed definitive therapy is a cornerstone of antimicrobial stewardship [5]. However, when cultures are negative, empiric antibiotics may be continued if there is an appropriate clinical response [4]. If there is no clinical response, the antibiotic regimen may be modified or escalated accordingly. Previous studies have defined empiric antibiotic treatment as antibiotics administered before pathogen identification by Gram staining or culture [6], or by other standard diagnostic tests such as the FilmArray Meningitis/Encephalitis Panel (FA-MEP) test [7]. The optimal choice and duration of empiric antibiotic therapy remain largely understudied, and there is considerable variation in antibiotic selection, dosing, and treatment duration for acute bacterial meningitis [1,4,8-11]. While international guidelines generally recommend treatment durations of 14-21 days for certain pathogens, the safety of abbreviating this duration in clinically stable patients remains an active area of investigation [12]. This review aimed to determine the comparative effectiveness of different durations of empiric antibiotic treatment in reducing mortality and morbidity among patients with suspected acute bacterial (non-tuberculous) meningitis.

## Review

Methods

We used the Preferred Reporting Items for Systematic Reviews and Meta-Analyses (PRISMA) guidelines to conduct the present review [13]. The PICO framework was used to define the objectives and formulate the search strategy (Table 1). The systematic review protocol was prospectively registered in the PROSPERO database (Registration Number: CRD42024612843).

**Table 1:** Detailed PICO framework *Patients who have been administered antibiotics (oral or parenteral) for 24 hours or more.

Framework	Description
Population	Patients with suspected bacterial (non-tuberculous) meningitis. Subgroups: (a) by age groups: neonates (<28 days), infants (>28 days to 1 year), children (1-18 years), adults (>18 to 50 years), and elderly (>50 years); (b) by special scenarios: pregnant women, postpartum women, patients who underwent neurosurgical procedures (including cochlear implants), patients with head injury, patients with immunocompromised conditions, and patients with prior use of antibiotics* (including partially treated pyogenic meningitis)
Interventions	Empiric antibiotics (single/combination of antibiotics) therapy for ≤10 days
Comparator	Empiric antibiotics (single/combination of antibiotics) therapy for 14-21 days
Outcome	Critical: (1) Mortality outcomes: all-cause mortality, case fatality rate, and survival rates (measured up to one year after discharge); (2) morbidity outcomes: short-term or long-term neurological complications (any motor, sensory (including hearing impairment), cognitive, or behavioral deficits, epilepsy, etc.) from discharge up to one year, any functional impairment (assessed by any validated scale at any time point) from discharge up to one year; (3) adverse effects of the antibiotic use. Important: (1) Hospitalization matrices: occurrence of seizures during hospitalization; (2) length of hospitalization; (3) time to resolution of symptoms

Search strategy

A systematic literature search was conducted across EMBASE, MEDLINE, SCOPUS, the Cochrane Central Register of Controlled Trials (CENTRAL), the Clinical Trials Registry-India (CTRI), and ClinicalTrials.gov, using the broad search terms outlined in Table 2.

**Table 2:** Search strategy

Database	Search details
EMBASE	empiric AND ("antibiotic agent"/exp OR "antibiotic agent") AND ("meningitis"/exp OR "meningitis")
COCHRANE	Three Cochrane Reviews matching Empiric antibiotics Meningitis in Title Abstract Keyword
PubMed	("empiric"[All Fields] OR "empirical"[All Fields] OR "empirically"[All Fields] OR "empirics"[All Fields]) AND ("anti bacterial agents"[Pharmacological Action] OR "anti bacterial agents"[MeSH Terms] OR ("anti bacterial"[All Fields] AND "agents"[All Fields]) OR "anti bacterial agents"[All Fields] OR "antibiotic"[All Fields] OR "antibiotics"[All Fields] OR "antibiotic s"[All Fields] OR "antibiotical"[All Fields]) AND ("meningeal"[All Fields] OR "meninges"[MeSH Terms] OR "meninges"[All Fields] OR "meninge"[All Fields] OR "meningism"[MeSH Terms] OR "meningism"[All Fields] OR "meningisms"[All Fields] OR "meningitis"[MeSH Terms] OR "meningitis"[All Fields] OR "meningitides"[All Fields]) AND 1000/01/01:2026/08/07[Date - Publication]
SCOPUS	TITLE-ABS-KEY (empiric AND antibiotics AND meningitis)
CTRI	No results
ClinicalTrials.gov	One result

Additionally, the reference lists of the included studies were reviewed to identify potentially relevant studies. Four investigators independently screened the abstracts, and selected articles underwent full-text evaluation. Any disagreements were resolved through consensus, resulting in the final list of included studies.

Eligibility criteria

Inclusion Criteria

RCTs and non-RCTs, as well as prospective and retrospective observational studies, that met the inclusion criteria and evaluated the use of empiric antibiotics for meningitis were included.

Exclusion Criteria

Case series, case reports, letters, editorials, commentaries, animal studies, and studies published in languages other than English were excluded.

Data extraction

**Figure 1:**
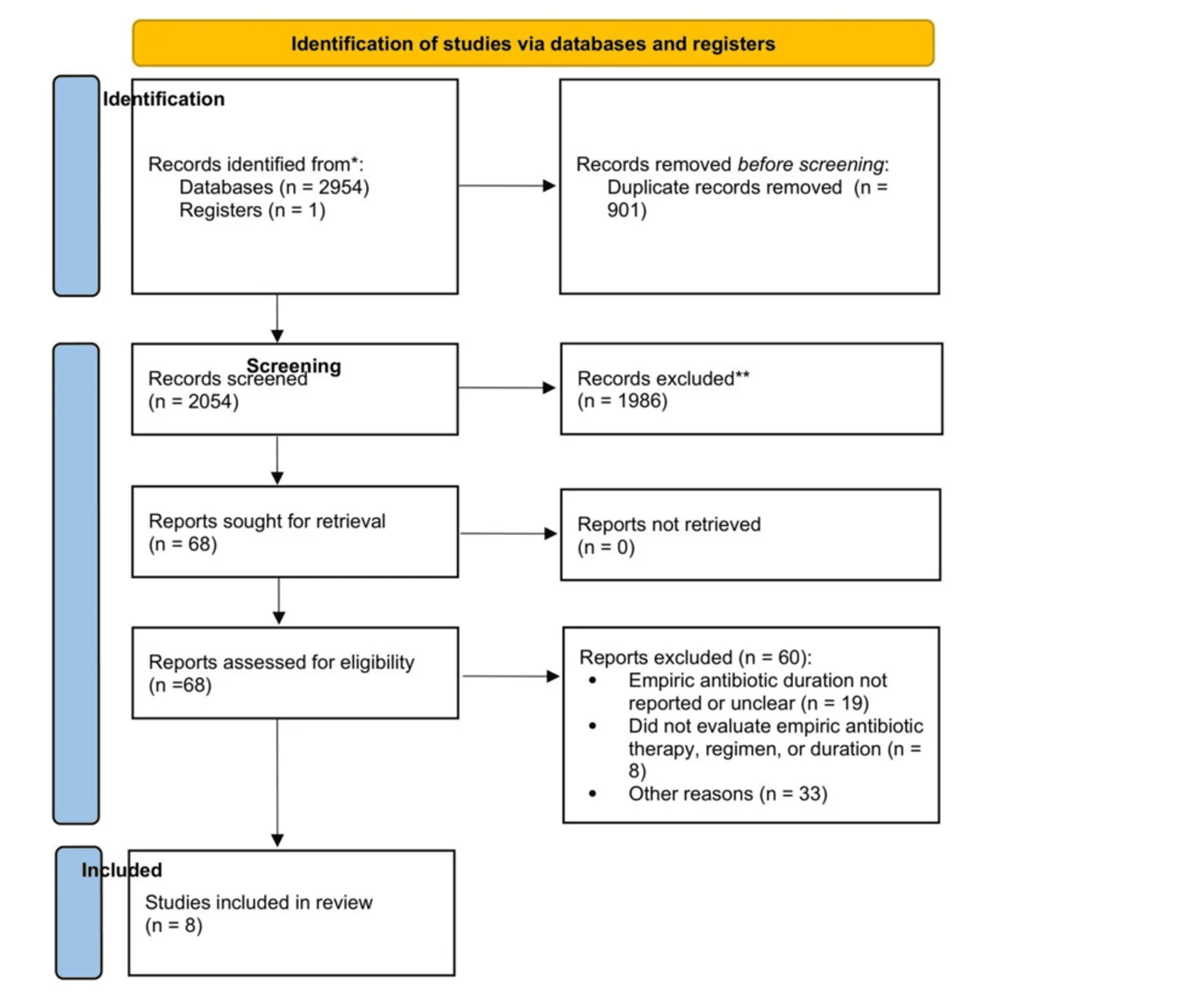
PRISMA flow diagram for new systematic reviews, which included searches of databases and registers only *Consider, if feasible to do so, reporting the number of records identified from each database or register searched (rather than the total number across all databases/registers). **If automation tools were used, indicate how many records were excluded by a human and how many were excluded by automation tools. Source: [13]. Note: This work is licensed under CC BY 4.0.

Two investigators independently assessed the included studies and extracted data using a predesigned proforma based on the inclusion criteria. The PRISMA flow diagram illustrating the study selection process is presented in Figure 1.

The extracted data included study ID, authors, year of publication, country, participant characteristics, inclusion criteria, sample size, age, sex, type of empiric antibiotic used, dosage and duration of treatment, reported outcomes, complications, and mortality. In cases where consensus could not be reached, a third senior investigator was consulted to make the final decision.

Missing data

Authors were contacted to obtain missing data. Any discrepancies or uncertainties were resolved through consultation and consensus among the investigators.

Risk of bias and quality assessment

We used the revised Cochrane Risk of Bias 2 (RoB 2) tool to assess the risk of bias in RCTs and the Joanna Briggs Institute (JBI) critical appraisal tool to assess non-RCTs and other studies, as appropriate.

Interpretation

The results were interpreted with caution, considering the potential limitations and biases inherent in the included studies. Due to substantial clinical heterogeneity among the trials with respect to antibiotic regimens and regional pathogen prevalence, a narrative synthesis approach was used in accordance with the Synthesis Without Meta-analysis (SWiM) reporting guidelines. The conclusions drawn from this review may provide valuable insights into the comparative effectiveness of short-course and standard-course antibiotic therapy and may aid evidence-based decision-making in clinical practice.

Study selection 

The search strategy outlined in Table 2 yielded 2,955 articles. After removing 901 duplicates, the remaining 2,054 articles were screened based on their titles and abstracts. Of these, 68 articles were retrieved for full-text review. Sixty articles [6,7,14-71] were excluded for the reasons provided in Table 3. The initial search was updated through July 2026 to include recently published contemporary studies, which were ultimately excluded because they did not provide direct comparative data on treatment duration.

**Table 3:** Studies not included and their reasons for exclusion

Study authors (year)	Reasons for exclusions
Brink et al. (2019) [14]	Although used empiric antibiotics but duration not clear
Buckingham et al. (2001) [15]	Culture-confirmed pneumococcal meningitis
Buckingham et al. (2006) [16]	Although used empiric antibiotics but duration not clear
Cabellos et al. (1999) [18]	No details of empiric antibiotics
Cabellos et al. (2022) [17]	No details of empiric antibiotics
Çay et al. (2023) [19]	Patients included with enterococcal meningitis/ventriculitis in children
Celal et al. (2004) [20]	The study does not focus specifically on empiric antibiotic therapy as an intervention
Choi et al. (2021) [21]	Retrospective pre-post study
Díaz et al. (2020) [23]	Includes meningitis and encephalitis separate data specifically for bacterial meningitis
D'Inzeo et al. (2020) [22]	Focuses on the diagnostic methods for bacterial meningitis rather than the duration of empiric antibiotic therapy
Eichinger et al. (2019) [24]	Although used empiric antibiotics but duration not clear
Elsaid et al. (2002) [25]	Although used empiric antibiotics but duration not clear
Elsaid et al. (2006) [26]	Although used empiric antibiotics but duration not clear
Fang et al. (2000) [27]	Full text not available
Fontela et al. (2017) [28]	Survey
Haddon et al. (1999) [29]	Does not evaluate specific empiric antibiotic regimens
Hasbun et al. (2017) [30]	Lacks details of empiric antibiotic regimens
Hasbun et al. (2019) [31]	Does not specifically describe empiric antibiotic therapy for suspected bacterial meningitis and duration
Houck and Rivera (2025) [32]	Abstract
Hussein et al. (2000) [33]	Although used empiric antibiotics but duration not clear
Jin et al. (2015) [34]	Does not focus on empiric antibiotic therapy
Karadag Oncel et al. (2020) [35]	Does not focus on empiric antibiotic therapy duration
Karageorgopoulos et al. (2009) [36]	Meta-analysis
Kellner et al. (2002) [37]	Although used empiric antibiotics but duration not clear
Khan (2021) [38]	Does not focus on empiric antibiotic therapy regimen and duration
Kizilates et al. (2021) [39]	Although used empiric antibiotics but duration not clear
Kruger et al. (2018) [40]	Study focuses on the use of empirical antibiotics in cases of viral meningitis rather than bacterial meningitis
Lee et al. (2026) [41]	Does not compare the clinical effectiveness of predefined treatment durations
McMaster et al. (2002) [42]	Although used empiric antibiotics but duration not clear
Messacar et al. (2022) [43]	Although used empiric antibiotics but duration not clear
Meyer et al. (2004) [6]	Although used empiric antibiotics but duration not clear
Mohialdin et al. (2024) [45]	Although used empiric antibiotics but duration not clear
Mohialdin et al. (2024) [44]	Evaluates guidelines use
Nicolau-Guillaumet et al. (2026) [46]	Does not have comparative control group for evaluating the efficacy of short vs. standard antibiotic courses
Olson et al. (2022) [47]	Poster abstract
Onita et al. (2024) [48]	No details related to empiric antibiotics
Paioni et al. (2020) [49]	Does not provide comparative data on treatment effectiveness
Park et al. (2021) [7]	Although used empiric antibiotics but duration not clear
Peterković et al. (2012) [50]	Studied the addition of dexamethasone to empiric antibiotic therapy in bacterial meningitis patients
Phi et al. (2022) [51]	Although used empiric antibiotics but duration not clear
Pintado, et al. (2020) [52]	Retrospective multicenter cohort study
Rafiei et al. (2025) [53]	Systematic review
Sáez-Llorens et al. (1995) [54]	Studied choice of antibiotics did not mention the duration
Saez-Llorens and O'Ryan (2001) [55]	Review
Sakai et al. (2011) [56]	Survey
Sidler et al. (2025) [57]	Does not evaluate clinical outcomes of fixed-duration short-course vs. standard-course treatment regimens
Sipahi et al. (2023) [58]	Although used empiric antibiotics but duration not clear
Smoke et al. (2020) [59]	Abstract
Spagnuolo et al. (1982) [60]	Case series, does not provide specific empiric antibiotic details
Sudo et al. (2024) [61]	Review article
Sulaiman et al. (2017) [62]	Although used empiric antibiotics but duration not clear
Tadesse et al. (2017) [63]	Although used empiric antibiotics but duration not clear
Tajdin et al. (2013) [64]	Although used empiric antibiotics but duration not clear
Tängdén et al. (2011) [65]	Although used empiric antibiotics but duration not clear
Terranella et al. (2012) [66]	Survey
Thampy et al. (2026) [67]	Meta-analysis
Thind et al. (2026) [68]	Abstract
Willey et al. (2024) [69]	Does not compare treatment durations
Zhang et al. (2026) [70]	Focuses on accuracy of the lab test
Zhao et al. (2019) [71]	Retrospective study

Study characteristics 

Eight studies [4,8-11,72-74], all of which were RCTs, were included in the qualitative synthesis (Table 4).

**Table 4:** Characteristics of the included studies

Author	Country	Type of Study	Target population	Sample size	Empiric antibiotic therapy	Remarks
Kavaliotis et al. (1989) [11]	Greece	RCT	Pediatric meningitis (>1 month)	52. Short course (n = 26): 4, 6, and 7 days for *Neisseria*/*Haemophilus* and *Streptococcus.* Standard course (26): 8, 12, and 14 days	Inj ceftriaxone 100 mg/kg loading dose followed by 60 mg/kg once daily	Small sample size, open-label RCT
Lin et al. (1985) [72]	USA	RCT	Pediatric meningitis (>1 month)	79. Seven-day group: n = 44. Ten-day group: n = 35	i.v. ceftriaxone, 75 mg/ kg once, followed by 50 mg/kg q12 h for 10 days (long) vs. 7 days (short duration)	Children with *Neisseria* were being treated with a 7-day course; only those with *Streptococcus* and *Haemophilus* were randomized for 7- and 10-day courses. One patient in the 7-day group received two extra doses of ceftriaxone due to associated septic arthritis
Martin et al. (1990) [10]	Switzerland	RCT	Pediatric meningitis (3 weeks to 15.5 years)	92. Short course (n = 47): 4, 6, and 7 days for *Neisseria* /*Haemophilus* and *Streptococcus* ​​​​​. Standard course (n = 45): 8, 12, and 14 days	Inj ceftriaxone 100 mg/kg/day once daily on D1 and D2, followed by 60 mg/kg once daily	Only patients with sterile CSF tap after 24 h of ceftriaxone. Patients with positive cultures are grouped in Group III (excluded). Neurological sequelae were more noticed in group III patients
Mathur et al. (2015) [73]	India	RCT	Neonatal bacterial meningitis	70. Short course: 10 days of antibiotics Standard: 14 days	Didn’t mention on specific antibiotic used; dexamethasone is used for adjuvant therapy	Blood/CSF culture is positive in only 6 patients in the short-course group vs. 7 in the standard-course group. Most common isolate was *Klebsiella,* with 66% sensitive to ceftriaxone, 75% to amikacin, and 50% to meropenem. Randomization done after D7
Molyneux et al. (2011) [8]	Bangladesh, Egypt, Malawi, Pakistan, and Vietnam	RCT	Pediatric meningitis (2 months to 12 years)	1,004. Short course (n = 496): 5 days. Standard course (n = 508): 10 days	Inj ceftriaxone (80-100 mg/kg) once daily. Dexamethasone given at some sites	Patients were excluded if the 2nd CSF sample at 48 h contains bacteria. Randomization was done at D5
Roine et al. (2000) [74]	Chile	RCT	Pediatric meningitis (>3 months)	102. Short course (n = 55): 4 days. Standard course (n = 47): 7 days	Inj ceftriaxone 100 mg/kg once daily for 4 days or 7 days with dexamethasone	Only patients with rapid initial recovery were included and randomized
Singhi et al. (2002) [9]	India	RCT	Pediatric meningitis (3 months to 12 years)	69. Short course (n = 35): 7 days. Standard course (n = 34): 10 days	Inj ceftriaxone 100 mg/kg/day in two divided doses	CSF cultures and microbiological testing results are limitations of this study
Vaswani et al. (2021) [4]	India	RCT	Pediatric meningitis (3 months to 14 years)	96. Short course (n = 48): 7 days. Standard course (n = 48): 14 days. Randomization done at D5	Inj ceftriaxone 100 mg/kg/day in two divided doses + vancomycin 60 mg/kg/day in four divided doses. Dexamethasone adjuvant therapy	Positive CSF gram stain D5 culture were excluded

The studies were published between 1985 and 2021. Three studies were conducted in India, while one study each was conducted in the USA, Greece, Switzerland, and Chile. One multinational study was conducted in Bangladesh, Egypt, Malawi, Pakistan, and Vietnam. Seven studies focused on pediatric meningitis, while one study addressed neonatal meningitis. None of the studies included adults. A total of 1,562 patients were included across all studies. Empiric antibiotic regimens varied across the studies. Studies published before 2011 predominantly used ceftriaxone monotherapy as empiric antibiotic therapy, with doses ranging from 50 to 100 mg/kg/day, administered in one or two divided doses. In contrast, studies published after 2011 used combination therapy with ceftriaxone and vancomycin. Dexamethasone was used as adjunctive therapy in four studies. The definition of short-course antibiotic therapy varied across the studies, ranging from 4 to 10 days. Bacterial cultures identified organisms including *Neisseria*, *Streptococcus*, *Haemophilus*, and *Klebsiella* species.

Effects of short course vs. standard course on neurological outcomes

The neurological outcomes reported across the studies included hearing loss, subdural effusion, abnormal brainstem-evoked response audiometry findings, hydrocephalus, developmental delay, convulsions, ventriculitis, and other neurological sequelae (Table 5).

**Table 5:** Reported outcomes in the included studies

Author	Non-neurological outcomes	Neurological outcomes	Hearing loss	CSF examination	Mortality	Others	Adverse effects
Kavaliotis et al. (1989) [11]	Persistent fever/pleocytosis (p > 0.1)	Neurological complications, 4 in the standard course (hearing loss, 3; p = 0.362; ataxia, 1; p = 1.5)	3 patients in the standard course have hearing loss	Cultures and smears were negative in both groups in all patients. 5 patients in the short course and one patient in the standard course had persistent CSF pleocytosis despite sterile CSF (19% vs. 3.8%; p = 0.139).	No deaths	No change in the treatment, 96% in the standard course vs. 81% in the short course (p = 0.139)	Diarrhea, 13.5% (p = 0.615)
Lin et al. (1985) [72]	Similar between the groups regarding nosocomial infections, associated infections, secondary fever, and surgery required for complications	Similar between the groups (11.4% in each group)	No difference; 70% in the 7-day group and 68% in the 10-day group have normal hearing	No significant difference between the 7 days and 10 days	No deaths	The 7-day group has significantly less hospitalization, with a mean of 8 days vs. 10 days in the standard group (p < 0.05)	Diarrhea due to ceftriaxone (suppression of gut bacteria) was reported in 17 patients of the 7-day group and 15 patients of the 10-day group
Martin et al. (1990) [10]	Prolonged or recurrent fever (26 children in group I and 13 in group II), aseptic arthritis, serum sickness syndrome	Neurological complications: subdural effusion, one in each group	NA	CSF examination after 15 h: negative in both groups (those with positive cultures were excluded and treated in group III)	No deaths	NA	Mild diarrhea, stomatitis, diaper rash/candidiasis
Mathur et al. (2015) [73]	Sepsis (n = 3) in the standard-course group (p = 0.24)	Abnormal brainstem-evoked response audiometry (BERA): one in the short-course group (p = 1.00)	NA	NA	1 in the short-course group and 2 in the standard-course group (p = 1.00)	NA	Not reported
Molyneux et al. (2011) [8]	NA	Visual loss, 1% in the short course vs. 2% in the standard course; motor deficits, 4% in the short course vs. 6% in the standard course; hydrocephalus, none in the short course vs. 1% in the standard course; developmental delay, 5% in the short course vs. 7% in the standard course (nonsignificant)	Hearing loss, 21% in the short course and standard course	NA	Deaths, 2% in the short course vs. 3% in the standard course (not significant)	Bacteriological failures, none in both groups	No adverse reactions reported
Roine et al. (2000) [74]	Persistent fever, 2 in the standard group vs. none in the short course	Subdural effusion, 1 in the short course; no significant difference with respect to convulsions (p = 0.94), readmission (p = 0.30), neurological sequelae (p = 0.39)	Auditory sequelae (p = 0.49)	NA	No deaths	Elevated CRP, 1 in the standard course vs. 2 in the short course	Not mentioned
Singhi et al. (2002) [9]	Nosocomial infections, 5.7% in the short course vs. 29.4% in the standard course (p < 0.05)	Subdural effusion, 8.6% in the short course vs. 20.6% in the standard course; ventriculitis, 5.7% in the short course vs. 2.9% in the standard course; no statistical significance	Hearing loss, speech delay, hypertonia, monoplegia, hemiplegia, and blindness were seen 25.7% in the short-course group vs. 44.1% in the standard course (no statistical significance)	NA	Short-course group: n = 1	Treatment failure based on a scoring system, 25.7% in the short course vs. 23.5% in the standard course (p-value not significant). Hospital stay, 10.8 ± 6.0 days in the short-course group and 14.2 ± 7.2 days in the standard course (p < 0.05)	Not mentioned
Vaswani et al. (2021) [4]	Nosocomial sepsis, two cases in each group	Hearing defects, hydrocephalus, and others, 11 patients in the short-course group vs. 9 patients in the standard course (no significant difference)	Mentioned in neurological complications	NA	No deaths	Treatment failures, 14.58% in the short course vs. 12.5% in the standard course (not significant)	No significant adverse effects of the drugs

No significant differences in neurological outcomes were observed across the studies. Kavaliotis et al. reported neurological complications in four patients in the standard-course group, including hearing loss in three patients (p = 0.362) and ataxia in one patient (p = 1.5) [11]. Lin et al. reported a similar incidence of neurological complications (11.4%) in both groups [72]. Normal hearing was reported in 70% of patients in the 7-day group and 68% of patients in the 10-day group, with no statistically significant difference between the groups [72]. Martin et al. reported subdural effusion in one patient in each group [10]. Mathur et al. reported abnormal brainstem-evoked response audiometry in one neonate in the short-course group (p = 1.00) [73]. Molyneux et al. reported visual loss in 1% versus 2%, motor deficits in 4% versus 6%, hydrocephalus in 0% versus 1%, and developmental delay in 5% versus 7% in the short-course versus standard-course groups, respectively [8]. Hearing loss was reported in 21% of patients in both the short-course and standard-course groups. Roine et al. reported subdural effusion in one patient in the short-course group, with no significant differences in the incidence of convulsions (p = 0.94), readmission (p = 0.30), or neurological sequelae (p = 0.39) [74]. Singhi et al. reported subdural effusion in 8.6% versus 20.6% of patients and ventriculitis in 5.7% versus 2.9% of patients in the short-course versus standard-course groups, respectively; neither difference was statistically significant [9]. Vaswani et al. reported no significant differences in hearing loss or hydrocephalus, with hydrocephalus occurring in 11 versus 9 patients in the short-course versus standard-course groups, respectively [4].

Effects of short course vs. standard course on non-neurological outcomes

Overall, no significant differences in non-neurological complications were reported by the included studies. Kavaliotis et al. reported persistent fever and pleocytosis, with p > 0.1 [11]. Lin et al. reported similar incidences of nosocomial infections, secondary fever, and the need for surgery due to complications between the two groups [72]. Martin et al. reported prolonged or recurrent fever in 26 versus 13 children in the short-course and standard-course groups, respectively [10]. The study also reported the incidence of septic arthritis and serum sickness syndrome, with no significant differences observed between the groups. Mathur et al. reported sepsis in three patients in the standard-course group (p = 0.24) [73]. Roine et al. reported persistent fever in two children in the standard-course group compared with none in the short-course group [74]. Singhi et al. reported nosocomial infections in 5.7% versus 29.4% of patients in the short-course versus standard-course groups, respectively, with a statistically significant difference (p < 0.05) [9]. Vaswani et al. reported two cases of nosocomial infection in each group [4].

Effects of short course vs. standard course on mortality

No mortality events were reported in five studies [4,10,11,72,74]. Mathur et al. reported one death in the short-course group compared with two deaths in the standard-course group (p = 1.00) [73]. Molyneux et al. reported mortality rates of 2% in the short-course group versus 3% in the standard-course group [8]. Singhi et al. reported one death in the short-course group [9]. Overall, these studies reported comparable clinical outcomes between the short-course and standard-course groups.

Effects of short course vs. standard course on other outcomes

Outcomes such as duration of hospitalization and treatment failure were reported in four studies. Lin et al. reported a significantly shorter duration of hospitalization in the short-course group compared with the standard-course group (p < 0.05) [72]. Singhi et al. also reported a significantly shorter hospital stay in the short-course group compared with the standard-course group (p < 0.05) [9]. Molyneux et al. reported no treatment failures in either group [8]. Singhi et al. reported treatment failure rates of 25.7% in the short-course group versus 23.5% in the standard-course group, with no statistically significant difference [9]. Vaswani et al. reported treatment failure rates of 14.58% in the short-course group versus 12.5% in the standard-course group, with no statistically significant difference [4].

Reported adverse effects of empirical antibiotics used

Seven studies reported adverse effects. Diarrhea was reported in 13.5% of patients in Kavaliotis et al. [11] and in 17 versus 15 patients in the short-course versus standard-course groups, respectively, in Lin et al. [72]. Martin et al. reported mild diarrhea, while the other studies reported no significant adverse effects [10]. Overall, no significant differences in adverse effects were reported between the short-course and standard-course groups. Other adverse effects reported included stomatitis and diaper rash/candidiasis [10].

Risk of bias of the included RCTs

The overall risk of bias varied across the included studies. Most of the potential bias was related to performance and detection bias, as most studies lacked blinding. Particularly in studies in which duration of hospitalization was an outcome measure, blinding may not have been feasible. Molyneux et al. addressed this issue by ensuring similar hospital stays in the intervention and control groups through the use of placebo injections [8]. A moderate-to-high risk of bias was observed in the study by Martin et al. [10], whereas the remaining studies were considered to have a moderate-to-low risk of bias.

Reasons for not doing a meta-analysis

Most of the studies published before 2011 used ceftriaxone monotherapy. At least two meta-analyses were conducted based on these studies. However, the findings of these earlier studies may not be fully applicable to the current clinical scenario, given the widespread emergence of antibiotic resistance among organisms such as *Streptococcus* and *Neisseria*. The microbial epidemiology of meningitis has also changed over time, with an increased incidence of newer causative organisms and the emergence of antimicrobial resistance. Most current guidelines recommend combination therapy, including vancomycin with ceftriaxone and/or other appropriate agents, for empiric antibiotic treatment. Only three studies included in the present review were published after 2011, of which one focused on pediatric meningitis and two focused on neonatal bacterial meningitis. Of the two studies involving neonatal meningitis, one was an RCT and the other was a retrospective study. A formal meta-analysis could not be performed because of substantial clinical heterogeneity across the studies, including variations in pathogen prevalence, regional antimicrobial resistance profiles, and the transition from monotherapy to combination therapy in more contemporary cohorts. Therefore, the lack of recent and uniform data limited our ability to perform an updated meta-analysis. Our review highlights the need for further RCTs to evaluate contemporary empiric antibiotic regimens and to support the development of evidence-based guidelines for the empiric treatment of bacterial meningitis.

Discussion

Bacterial meningitis continues to pose a significant global health challenge, requiring prompt diagnosis and appropriate antibiotic therapy to reduce mortality and morbidity. The selection and duration of antibiotic therapy depend on the causative pathogen, host factors, and local antimicrobial resistance patterns. Current clinical guidelines emphasize pathogen-directed antibiotic therapy, which can optimize treatment effectiveness while helping to limit the development and spread of antimicrobial resistance (Table 6).

**Table 6:** Antibiotic duration guidelines for meningitis

Guideline	Pathogen	Pediatric duration	Adult duration
Indian Academy of Pediatrics (IAP) Standard Treatment Guidelines [75]	Neisseria meningitidis	5-7 days	5-7 days
	Haemophilus influenzae	7-10 days	7-10 days
	Streptococcus pneumoniae	10-14 days	10-14 days
	Gram-negative bacilli	21-28 days	21-28 days
Infectious Diseases Society of America (IDSA) Guidelines [12]	Neisseria meningitidis	7 days	7 days
	Haemophilus influenzae	7 days	7 days
	Streptococcus pneumoniae	10-14 days	10-14 days
	Listeria monocytogenes	≥21 days	≥21 days
Médecins Sans Frontières (MSF) Medical Guidelines [76]	General bacterial pathogens	10 days	10 days
European Federation of Neurological Societies (EFNS) Guidelines [77]	Neisseria meningitidis	4-7 days	4-7 days
	Haemophilus influenzae	7-10 days	7-10 days
	Streptococcus pneumoniae	10-14 days	10-14 days
	Listeria monocytogenes	≥21 days	≥21 days
World Health Organization (WHO) Guidelines [78]	Neisseria meningitidis	5-7 days	5-7 days
	Haemophilus influenzae	7-10 days	7-10 days
	Streptococcus pneumoniae	10-14 days	10-14 days
Canadian Paediatric Society [79]	Streptococcus pneumoniae	10-14 days	Not specified
	Haemophilus influenzae	7-10 days	Not specified
	Neisseria meningitidis	5-7 days	Not specified
	Group B *Streptococcus*	14-21 days	Not specified
German Society for Neurology [80]	Haemophilus influenzae	7-10 days	7-10 days
	Streptococcus pneumoniae	10-14 days	10-14 days
UpToDate [81]	Neisseria meningitidis	Not specified	7 days
	Streptococcus pneumoniae	Not specified	10-14 days
	Listeria monocytogenes	Not specified	≥21 days
Medscape [82]	Neisseria meningitidis	7 days	7 days
	Haemophilus influenzae	7 days	7 days
	Streptococcus pneumoniae	10-14 days	10-14 days
	Listeria monocytogenes	≥21 days	≥21 days

Neisseria meningitidis is one of the major bacterial pathogens causing meningitis worldwide. Major guidelines recommend treatment with ceftriaxone or penicillin G for 5-7 days [60-64]. Evidence suggests that treatment courses of less than 7 days may be effective for uncomplicated meningitis in immunocompetent patients. For *Haemophilus influenzae *meningitis, ceftriaxone or cefotaxime is generally recommended for 7-10 days to achieve effective treatment and reduce the risk of relapse [61-66].

Treatment of *Streptococcus pneumoniae* meningitis generally requires ceftriaxone or cefotaxime for 10-14 days, with the addition of vancomycin when antimicrobial resistance is suspected or confirmed [61,64,66,68]. An extended treatment duration of 10-14 days is recommended because of the higher risk of neurological complications associated with pneumococcal meningitis. For *Listeria monocytogenes* meningitis, particularly in neonates, pregnant women, and immunocompromised patients, ampicillin or penicillin G is generally administered for at least 21 days [63,67,68]. Gentamicin may be added in severe infections to provide potential synergistic antimicrobial activity.

Current research continues to clarify how treatment effectiveness can be optimized while adhering to antimicrobial stewardship principles. Recent evidence suggests that selected patients may be successfully treated with abbreviated antibiotic regimens, which can shorten hospital stays and reduce drug-related toxicity and antimicrobial resistance [62,65,67]. Pneumococcal meningitis generally requires a longer treatment duration, particularly in high-risk patients and those with complications [61,63,68]. Advances in rapid diagnostic tools, such as polymerase chain reaction (PCR) and next-generation sequencing, have substantially improved pathogen detection and antimicrobial resistance profiling. These diagnostic tools enable clinicians to initiate pathogen-directed antibiotic therapy earlier, thereby potentially reducing the need for nonspecific broad-spectrum antibiotics [32,64,66]. The use of corticosteroids as adjunctive therapy with antibiotics in pneumococcal meningitis has also gained considerable interest, as studies have demonstrated improved outcomes with corticosteroid therapy in selected patients [65,67].

Future research should focus on establishing optimal treatment durations, developing individualized treatment strategies, and maximizing clinical benefits through effective antimicrobial stewardship. Monitoring antimicrobial resistance patterns and developing novel therapeutic agents will play an important role in improving patient outcomes. Rapid diagnostic tools, evidence-based changes in clinical practice, and global health initiatives will be essential for advancing the management of bacterial meningitis. This systematic review was conducted to inform the development of evidence-based recommendations for empiric antibiotic therapy for meningitis in children, adults, and special populations in the Indian context. Antibiotic treatment of meningitis dates back to the 1930s, when sulfonamides were used, and subsequently evolved to include chloramphenicol and penicillin in the 1950s [1]. With the emergence of antimicrobial resistance and the superior cerebrospinal fluid penetration of third-generation cephalosporins, ceftriaxone became a preferred antibiotic for the treatment of bacterial meningitis [61,64]. The relative frequency of bacterial pathogens causing community-acquired, non-tuberculous bacterial meningitis has also changed over the decades. *Haemophilus influenzae* was previously among the most common causes of bacterial meningitis, followed by *Streptococcus pneumoniae* and *Neisseria meningitidis* [2,63]. With widespread immunization, the incidence of *Haemophilus influenzae* meningitis has decreased, while *Streptococcus pneumoniae* has become a more prominent causative pathogen. *Listeria monocytogenes* has also emerged as an important cause, particularly in specific high-risk populations [64]. The emergence of penicillin resistance in *Streptococcus pneumoniae* has led to the use of combination therapy with ceftriaxone and vancomycin [61,67]. The duration of antibiotic therapy has also evolved over time, from more than 2-3 weeks initially to less than 10 days for many bacterial pathogens, with certain exceptions [32].

Our findings suggest that shorter antibiotic treatment durations resulted in outcomes comparable to those of standard-duration therapy with respect to neurological and non-neurological outcomes [4,8,9]. The short-course durations in the included studies ranged from 7 days in Lin et al. [72], Singhi et al. [9], and Vaswani et al. [4], to 5 days in Molyneux et al. [8] and 4 days in Roine et al. [74]. Kavaliotis et al. [11] and Martin et al. [10] evaluated different treatment durations according to the causative pathogen, with 4, 6, and 7 days for *Neisseria*, *Haemophilus*, and *Streptococcus*, respectively. Mathur et al., who focused on neonatal meningitis, defined the short-course duration as 10 days [73]. The other studies used ceftriaxone monotherapy, whereas Vaswani et al. [4] used a combination of ceftriaxone and vancomycin. Mathur et al. did not specify the antibiotic regimen used [73]. Regarding non-neurological outcomes, nosocomial infections were significantly more frequent in the standard-course group in the study by Singhi et al. [9]; otherwise, the reported non-neurological outcomes were comparable between the groups. Neurological outcomes, including hearing loss, as well as mortality, were also comparable between the short-course and standard-course groups across the studies. The duration of hospitalization was significantly shorter in the short-course groups in the studies by Lin et al. [72] and Singhi et al. [9]. Overall, these findings support consideration of short-course antibiotic therapy as a potential alternative to standard-course therapy in selected patients with bacterial meningitis.

A shorter duration of antibiotic therapy may reduce the duration of hospitalization and associated healthcare costs while also addressing concerns regarding antimicrobial resistance. Our findings are consistent with several current clinical guidelines [75,78]. However, these results should be interpreted with caution because of heterogeneity in the inclusion criteria across the included studies. In addition, most of the studies were published before 2011 and had relatively small sample sizes. Although a recent meta-analysis by Sudo et al. [61] also examined treatment durations in pediatric meningitis, our review specifically focused on the empiric phase of therapy and highlights landmark randomized evidence from diverse clinical settings. The included studies primarily focused on pediatric meningitis; none evaluated adults or special populations, such as pregnant women or patients with previous cranial surgery. The risk of bias in the included studies was also a concern, as blinding was not feasible in many cases. Despite these limitations, our review highlights the need for well-designed RCTs with standardized antibiotic durations, at least within specific regions, and standardized antibiotic regimens to support the development of evidence-based guidelines rather than reliance on individual clinical experience in the management of non-tuberculous bacterial meningitis [36]. A particular consideration is neonatal meningitis. Among the studies included in our review, antibiotic treatment durations for neonatal meningitis were longer than those used for pediatric meningitis [71,73]. This difference may be explained by the fact that neonatal bacterial meningitis is frequently associated with vertical transmission or hospital-acquired infection and has a relatively high incidence of Gram-negative bacterial pathogens.

Limitation

Empiric antibiotics are generally administered until the culture results become available, which usually takes approximately 2-3 days, after which definitive, pathogen-directed antibiotic therapy can be initiated. However, empiric antibiotics may be continued when cultures are negative, particularly when the clinical response supports the diagnosis of bacterial meningitis. Therefore, the duration of antibiotic therapy may be considered independently of the duration of empiric therapy. Shorter overall antibiotic treatment durations could reduce the duration of hospitalization and overall antibiotic exposure without compromising treatment outcomes. However, this review has several limitations. A primary limitation is the focus on pediatric and neonatal populations, as no adult RCTs met the strict inclusion criteria for comparative evaluation of treatment duration. The single-center design, small sample size, which was lower than the calculated sample size because of resource constraints, and exclusion of severe cases limit the generalizability of the findings. Additionally, the lack of advanced diagnostic tools, such as PCR or antigen testing, meant that the bacterial etiology could not be confirmed in all cases. This was particularly relevant because many children had received antibiotics before admission, potentially resulting in negative culture results. The impact of adjunctive therapies, such as dexamethasone, on treatment outcomes was also not evaluated. Despite these limitations, the findings support the potential use of shorter antibiotic courses in carefully selected pediatric patients and underscore the need for further multicenter research to validate these findings and develop evidence-based guidelines for the management of bacterial meningitis [4].

## Conclusions

This review supports the use of short-course antibiotic therapy as a safe and effective alternative to standard-course regimens for pediatric and neonatal meningitis. However, the lack of homogeneity in antibiotic selection and treatment duration, together with limited contemporary evidence, highlights the need for well-designed RCTs to further evaluate short-course therapy in the current clinical context. Future research should focus on establishing standardized, evidence-based guidelines for antibiotic selection, dosing, treatment duration, and the use of adjunctive therapies in the management of bacterial meningitis.
